# Recent Origin of a Range‐Restricted Species With Subsequent Introgression in its Widespread Congener in the *Phyteuma spicatum* Group (Campanulaceae)

**DOI:** 10.1111/mec.17624

**Published:** 2024-12-13

**Authors:** Dennis Larsson, Petra Šarhanová, Ovidiu Paun, Gerald M. Schneeweiss

**Affiliations:** ^1^ Department of Botany and Biodiversity Research University of Vienna Vienna Austria; ^2^ Department of Botany and Zoology Masaryk University Brno Czech Republic

**Keywords:** coalescent modelling, dichotomous versus hybridogenic origin, French massif central, phylogeography, range‐restricted endemics

## Abstract

Understanding the causes of restricted geographic distributions is of major interest to evolutionary and conservation biologists. Inferring historical factors has often relied on *ad hoc* interpretations of genetic data, and hypothesis testing within a statistical framework under different demographic scenarios remains underutilised. Using coalescent modelling on RAD‐sequencing data, we (i) test hypotheses about the origin of *Phyteuma gallicum* (Campanulaceae), a range‐restricted endemic of central France sympatric with its widespread congener *Ph. spicatum*, and (ii) date its origin, irrespective of its mode of origin, to test the hypothesis that the restricted range is due to a recent time of origin. The best supported model of origin is one of a dichotomous split of *Ph. gallicum*, confirmed as distinct species, and the Central European *Ph. nigrum* with subsequent gene flow between *Ph. gallicum* and *Ph. spicatum*. The split of *Ph. gallicum* and *Ph. nigrum* is estimated at 45–55,000 years ago. Coalescent modelling on genomic data not only clarified the mode of origin (dichotomous speciation instead of a previously hypothesised hybridogenic origin) but also identified recency of speciation as a sufficient, although likely not the sole, factor to explain the restricted distribution range. Coalescent modelling strongly improves our understanding of the evolution of range‐restricted species that are frequently of conservation concern, as is the case for *Ph. gallicum*.

## 1 Introduction

Plant species with restricted geographic distributions have been extensively studied to better understand the factors that determine their range limitation (Murray et al. 2002; Lavergne et al. 2004; Espeland and Emam 2011; Cardillo, Dinnage, and McAlister 2019; Sheth, Morueta‐Holme, and Angert 2020), but only rarely against widespread relatives. Apart from the ecological and evolutionary interest in the topic, the gained knowledge is useful for conservation biologists to devise long‐term strategies for management actions (Gaston and Kunin 1997), which have recently become crucial in the face of ongoing environmental challenges that may cause areas of range‐restricted species to shrink, possibly resulting in their extinction (Gibson et al. 2010; Casazza et al. 2014).

The factors that determine a species' range can roughly be divided into ecological and historical ones. Ecological factors concern current interactions between plants and their environment, such as competition for resources, including pollinators (Karron 1987), or tolerance for abiotic conditions (Lavergne et al. 2004), and the presence of geographical barriers to dispersal. Historical factors include past climatic fluctuations, human activity or time since origin (Simurda, Marshall, and Knox 2005; Coppi, Mengoni, and Selvi 2008; Casazza et al. 2013; Pouget et al. 2013; Sheth, Morueta‐Holme, and Angert 2020). Unlike ecological factors, which can be experimentally tested, historical factors can only be inferred from reconstructions. This has traditionally been done using *ad hoc* interpretations (i.e., interpretative phylogeography *sensu* Larsson, Pan, and Schneeweiss 2021) of patterns in genetic data inferred via, for instance, phylogenetic trees and networks, hierarchical clustering of population structure, demographic analyses (e.g., Bayesian Skyride plot and bottleneck tests) and, more rarely, results from species distribution modelling and niche similarity tests (Simurda, Marshall, and Knox 2005; Coppi, Mengoni, and Selvi 2008; Marcussen et al. 2011; Mayol et al. 2012; Casazza et al. 2013; Pouget et al. 2013; Gargiulo et al. 2019; Grünig, Fischer, and Parisod 2021). Present demographic and geographic distribution patterns may, however, be explained by more than one type of historical event (Mayol et al. 2012; Casazza et al. 2013), whose plausibilities cannot be assessed by interpretative phylogeography. Such testing can be achieved by comparing models simulating the hypothesised events, using, for instance, coalescent modelling, within a statistical framework such as maximum likelihood or Bayesian Inference (i.e., statistical phylogeography: Knowles and Maddison 2002).

Hypothesis testing within a statistical framework under scenarios involving complex demographic events (bottlenecks and gene flow, including timing of events) has been successfully done in widespread species (Bagley et al. 2017; González‐Martínez, Ridout, and Pannell 2017; Lu et al. 2019, 2020; Ye et al. 2020), but such an approach is expected to work well also in range‐restricted species. Indeed, statistical phylogeography has already been applied to range‐restricted plants (Douglas et al. 2011; Fernández‐Mazuecos and Vargas 2013; Shang et al. 2015; Tournebize et al. 2017; Liu et al. 2021). Whereas the power to distinguish models was too low when using small datasets (Douglas et al. 2011; Fernández‐Mazuecos and Vargas 2013; but see Shang et al. 2015), this can be alleviated by using NGS data (Tournebize et al. 2017; Liu et al. 2021), such as RADseq data (Davey and Blaxter 2010; Emerson et al. 2010; Hohenlohe et al. 2010; Andrews et al. 2016).

**FIGURE 1:**
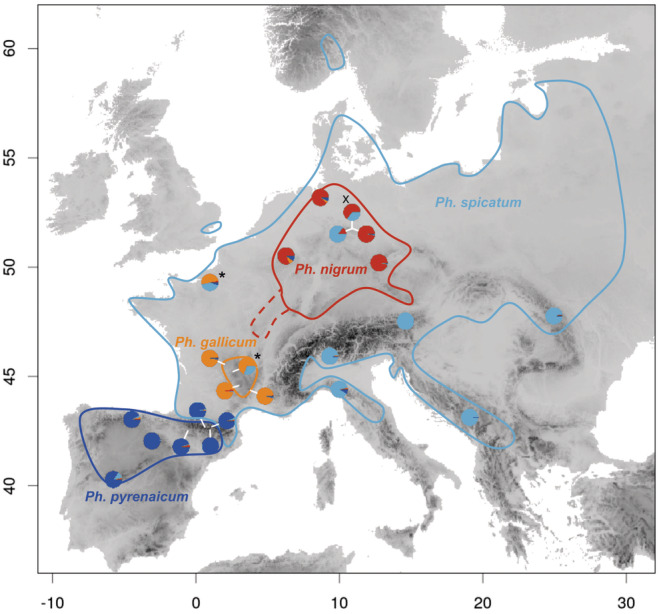
Distribution ranges of *Ph. gallicum* and related species (uncertain parts of the distribution range of *Ph. nigrum* indicated by dashed lines) and genetic groups inferred from RADseq data. The pie charts show the average ancestry coefficients of each population, the colours corresponding to the four species; some of the pie charts are displaced for better visualisation, and are connected to the actual sampling site by a white line. The pie chart with an x (northern Germany) indicates *Ph. × adulterinum*, and those with asterisks (France) western *Ph. spicatum*.

A good system to infer historical causes for range restriction using coalescent modelling is *Phyteuma gallicum* (Campanulaceae), a species restricted to the northern and western parts of the French Massif Central (henceforth referred to as the Atlantic Massif Central). Here, it occurs sympatrically with the closely related congener *Ph. spicatum* (Schneeweiss et al. 2013), which shares morphological and ecological traits with *Ph. gallicum* (Schulz 1904; Brunerye 1989), but occupies a much broader range in Europe (Figure 1). Because there is no evidence of ecological factors that would restrict *Ph. gallicum* to the Atlantic Massif Central, it can be hypothesised that historical factors in general and recency of speciation in particular are (co‐)responsible for the restricted range of this species. Speciation during the Quaternary was, in contrast to traditional beliefs (Willis and Niklas 2004; Bennett 2004, 2013), rather frequent (Nevado et al. 2018; Escudero et al. 2019; Vargas et al. 2020; Otero, Fernández‐Mazuecos, and Vargas 2021; see review by Kadereit and Abbott 2021), and taxa (nearly) restricted to the Atlantic Massif Central, such as *Arabidopsis cebennensis* (Brasssicaceae) or *Leucanthemum monspeliense* (Asteraceae), appear to be no exception (Koch, Wernisch, and Schmickl 2008; Konowalik et al. 2015; Jacquemin et al. 2016).

Here, we investigate the mode and time of origin of *Ph. gallicum* and assess recency of speciation as a potential historical factor for range restriction in this species. Using coalescent modelling on RAD‐sequencing data, we want (i) to test competing hypotheses about the origin of *Ph. gallicum* as a split off from Central European *Ph. nigrum*, as suggested by Schulz (1904), versus it being of hybridogenic origin involving *Ph. nigrum* and *Ph. spicatum*, as suggested by Schneeweiss et al. (2013) based on conflicting phylogenetic positions inferred from nuclear versus plastid sequence data; and (ii) to date the origin of *Ph. gallicum*, irrespective of its mode of origin, as a recent origin has been suggested as a cause for area limitation for other endemics in the Atlantic Massif Central (e.g., Koch, Wernisch, and Schmickl 2008; Konowalik et al. 2015; Jacquemin et al. 2016; Fabritzek, Griebeler, and Kadereit 2021).

## 2 Materials and Methods

### 2.1 Study Species

The focal species *Ph. gallicum* is a forb growing in mountain hay meadows and edges of montane forests (Schulz 1904; Brunerye 1989; Tison and de Foucault 2014). Phytosociologically, it is a character species of the alliance *Polygono bistortae–Trisetion flavescentis* (syn. *Triseto flavescentis–Polygonion bistortae*), comprising mesophilous mountain hay meadows (Tela Botanica 2023). Although this habitat is widespread in Central European mountain ranges, the species is restricted to the Massif Central in southeastern France. There are no known ecological factors that would constrain the distribution of *Ph. gallicum* to the Massif Central (Schulz 1904; Damboldt 1976; Brunerye 1989; Tison and de Foucault 2014), detailed ecological studies on *Ph. gallicum* are, however, lacking. Whereas it is considered ‘Least concern’ in the former region Auvergne, it is considered ‘Vulnerable’ in the former region Limousin and ‘Near threatened’ in the former region Midi‐Pyrénées (MNHN and OFB 2023).

The distribution range of *Ph. gallicum* is entirely nested within that of *Ph. spicatum*, which is widespread in Europe (Schulz 1904; Meusel and Jäger 1992; Figure 1). Although populations from the Iberian Peninsula and the Pyrenees are commonly subsumed under *Ph. spicatum* (Sales and Hedge 2000), these are phylogenetically clearly separated (Schneeweiss et al. 2013; this study) and are treated (following Schulz 1904, and Meusel and Jäger 1992) as *Ph. pyrenaicum*. As in its entire range, in the Massif Central, *Ph. spicatum* is mostly associated with forests and woods, whereas *Ph. gallicum* has a stronger affinity to meadows (Brunerye 1989). Morphologically, *Ph. spicatum* differs from *Ph. gallicum* in features of the basal leaves (maximally twice as long as broad with a strongly cordate base, often with black stains vs. usually more than twice as long as broad with weekly cordate to obtuse base, never with black stains) and the inflorescences (up to 12 cm long, cylindrical, with white, rarely pale or dark blue flowers vs. rarely more than 2 cm long, ovate, with sky‐blue flowers: Brunerye 1989). Where the two species meet, often along forest edges at intermediate altitudes, they are reported to frequently hybridise and form hybrid swarms (Brunerye 1989), but the hybrid nature has been inferred exclusively from morphological characters. A situation similar to that in *Ph. gallicum* is found for *Ph. nigrum*, presumably the closest relative of *Ph. gallicum* (Schulz 1904). The distribution range of *Ph. nigrum*, covering western Central Europe (Meusel and Jäger 1992; Figure 1), is entirely nested within that of *Ph. spicatum*, and the two species are known to hybridise (Schulz 1904; Weeda 1989; Buttler and Hand 2008; Müller et al. 2021). Morphologically, *Ph. gallicum* can be distinguished from *Ph. nigrum* by smaller and denser foliage, basal leaves that are already withered at anthesis, azure blue instead of purplish black (rarely blue) corollas and different shape of the lower cauline leaves (Schulz 1904).

### 2.2 Sampling

Leaf material along with voucher specimens from several sampling sites for each study taxon were collected in the field and stored in silica gel (Table 1). In addition to *Ph. gallicum*, *Ph. nigrum* and *Ph. spicatum*, we also included *Ph. pyrenaicum*, which is geographically close to *Ph. gallicum* and thus may have been involved in its origin (*Ph. pyrenaicum* was represented only by a single specimen in Schneeweiss et al. 2013), and *Ph. × adulterinum*, a known hybrid between *Ph. spicatum* and *Ph. nigrum*, as a reference for how a hybrid behaves in the analyses. Since unbalanced sampling is an issue in STRUCTURE‐like analyses (Puechmaille 2016), the widespread *Ph. spicatum* was subsampled from a larger dataset: First the population structure of *Ph. spicatum* was analysed separately (data not shown), and then two sampling sites from each of the four identified phylogeographic groups (western Europe; Apennines and southern Alps; eastern Alps and Balkans; central and eastern Europe) were selected. Thus, *Ph. spicatum* exceeded the second‐most numerous species, *Ph. pyrenaicum*, only by one sampling site (and by three individuals).

**TABLE 1:** Sampled taxa, sampling localities (country and coordinates), individual count per locality (*n*), and voucher information.

Taxon/Sample	Country	Latitude	Longitude	*n*	Voucher information
*Phyteuma × adulterinum*					
Site 1	Germany	10.887778	51.761111	4	Reich et al. (2017) (WU 0093668)
*Phyteuma gallicum*					
Site 1	France	1.974444	45.580833	3	Weis (2017) (GMS‐128: WU 0158441)
Site 2	France	3.838056	44.352500	4	Weis (2017) (GMS‐137: WU 0158443)
Site 3	France	3.025000	44.595278	4	Weis (2017) (GMS‐141: WU 0158439)
*Phyteuma nigrum*					
Site 1	Czech Republic	12.765470	50.194300	4	Hanzl (2012) (CP 531: voucher lost)
Site 2	Germany	6.285833	50.514722	4	Reich and Sander (2017) (WU 0093677)
Site 3	Germany	10.887778	51.761111	4	Reich et al. (2017) (WU 0093664)
Site 4	Germany	8.675000	53.188056	4	Schultz (2017) (GMS‐160: WU 0158453)
*Phyteuma pyrenaicum*					
Site 1	France	1.160359	42.720866	4	Escobar García (2017) (2833/2017: WU 0158450)
Site 2	Spain	0.620850	42.683622	4	Escobar García (2017) (2849/2017: WU 0158451)
Site 3	Spain	−4.457256	43.034257	4	Escobar García (2017) (3004/2017: WU 0158452)
Site 4	Spain	−5.740833	40.285000	3	García Muñoz (2012) (CP585: WU 0070838/WU 0070839)
Site 5	Spain	−0.528118	42.526622	4	Escobar García (2018) (P605/2018: WU 0158437)
Site 6	Spain	1.013446	42.581069	4	Escobar García (2018) (P613/2018: WU 0158438)
Site 7	Spain	−3.059224	42.050495	4	Escobar García (2018) (P621/2018: WU 0158436)
*Phyteuma spicatum*					
Site 1	Italy	9.333500	45.926694	3	Pachschwöll (2011) (CP5: WU 0062129)
Site 2	Italy	9.996111	44.406111	4	Reich and Hofbauer (2017): (WU 0094819)
Site 3	Montenegro	19.086111	43.133056	4	Weis and Thalinger (2013) (CP 969: WU 0087204)
Site 4	Austria	14.607500	47.526944	4	Ertl (2017) (GMS‐171: WU 0158444–WU 0158449)
Site 5	Germany	10.887778	51.761111	4	Reich et al. (2017) (WU 0093667)
Site 6	Ukraine	24.961736	47.770869	4	Pachschwöll and Pochynok (2017) (CP1154: WU 0158435)
Site 7	France	0.978889	49.295278	3	Weis (2017) (GMS‐122: WU 0158440)
Site 8	France	2.571944	45.220556	4	Weis (2017) (GMS‐130: WU 0158442)

### 2.3 DNA Extraction and RADseq

DNA was extracted using the Invisorb Spin Plant Mini Kit (Invitec Molecular, Berlin, Germany) following the manufacturer's protocol with one modification: After the lysis of the leaf material, 200 μL of chloroform:isoamyl alcohol (24:1) was added to the lysis solution, centrifuged for 5 min at 11,000 rpm and only the upper phase was transferred to the prefilter. The genomic extracts were cleaned with NucleoSpin gDNA Clean‐up (Macherey‐Nagel, Düren, Germany) following the manufacturer's instructions and eluted in 100‐μL ddH_2_O. DNA concentrations of all samples were estimated on a Qubit Fluorometer (Thermo Fisher Scientific, Waltham, USA).

Preparation of RAD libraries followed Paun et al. (2016) except using PstI for digestion, 150 ng of DNA as starting material and taking 72 individuals per library. A final size selection was done with a Pippin Prep (Sage Science, Beverly, MA, USA) targeting the size range 250–850 bp. Libraries were sequenced on the Illumina HiSeq2000 v4 platform using single‐end 100 bp reads at the Next Generation Sequencing Facility at the Vienna BioCenter Core Facilities (VBCF), member of the Vienna BioCenter (VBC), Austria (https://www.viennabiocenter.org/vbcf).

### 2.4 Assembly and Demultiplexing

The reads were demultiplexed and quality filtered using *BamIndexDecoder* from *illumina2bam 1.03* (https://github.com/gq1/illumina2bam) using default settings and *process_radtags* from *Stacks2 2.41* (Rochette, Rivera‐Colón, and Catchen 2019) with default settings, except a stricter read filtering by setting the sliding window size to be 6% (instead of the default 15%) of the length of the read and increasing the phred score limit from 10 to 20.

To find the optimal assembly settings for the *denovo_map.pl* script from *Stacks2*, we followed Paris, Stevens, and Catchen (2017) and explored, in a subsampled dataset consisting of the individual with the highest number of reads from each sampling site, different values for the two parameters *M*, the allowed differences between alleles within individuals and *n*, the allowed differences between loci across individuals. First, the diagonal of the parameter space was explored (*M* = *n* = 1, *M* = *n* = 2, etc.) up to a value of 7, then the *n* value for the best *M* parameter setting was explored (*M* = 4, *n* = 1; *M* = 4, *n* = 2; etc.), also up to a value of 7. The assembly parameters that produced the highest number of r80 loci (i.e., loci present in at least 80% of individuals) were selected.

Following Brandrud et al. (2020), we retained polymorphic RADtags with a maximum of 60% missing data across individuals and a maximum of 10 SNPs per locus using a combination of *populations* from *Stacks2* and a custom script. Additionally, we retained only loci identified as belonging to a spermatophyte by blasting the RADtags against the BLAST databases (downloaded 7th June 2019) *nt*, *env_nt*, *est_human*, *htgs* and *sts*, using *blastn* from *BLAST+ 2.9.0* (Camacho et al. 2009) and custom scripts.

As in Brandrud et al. (2020), we further mapped the raw reads of all individuals against an artificial reference including the filtered RADtags using *Bowtie2 2.3.4.1* (Langmead and Salzberg 2012). The SAM files were converted to BAM files, sorted by reference coordinates and read groups were added using *Picard 2.20.1* (http://broadinstitute.github.io/picard/). Realignments around indels were done using *Genome Analysis Toolkit 3.8* (GATK; McKenna et al. 2010).

Final genotypes were called using *ref_map.pl* from *Stacks2*. The SNPs were filtered into two datasets: one, for exploratory purposes, comprising all investigated taxa including *Ph. pyrenaicum* and *Ph. × adulterinum* (hereinafter the ‘complete dataset’); and a second one, for coalescent modelling, comprising only samples from *Ph. gallicum, Ph. nigrum* and *Ph. spicatum* (hereinafter the ‘reduced dataset’). The complete dataset was filtered as follows: For each locus with at most 10 SNPs, a single random SNP with at most 50% missing data, a maximum observed heterozygosity of 50% and that was variable in more than one individual (i.e., not a singleton) was extracted using *populations* from *Stacks2* (Han, Sinsheimer, and Novembre 2014; O'Leary et al. 2018). The reduced dataset was further filtered by only retaining SNPs from loci with a coverage of at least six using *vcftools 0.1.15* (Danecek et al. 2011) and by requiring that all SNPs must be present in at least two individuals per sampling site (Han, Sinsheimer, and Novembre 2014; O'Leary et al. 2018). The former aims to filter out loci where the alternative allele is likely to be missing due to low read count (Arnold et al. 2013), thus obtaining more accurate estimates of homozygosity, and the latter to ensure that every sampling site is represented by at least four (haploid) genotypes in the dataset. Finally, loci that had an allele frequency of exactly 0.5:0.5 over the entire dataset were removed since their minor allele frequency cannot be determined.

### 2.5 Data Analyses

To reconstruct the potentially reticulate relationships between the taxa, a neighbour net was calculated using the Hasegawa–Kishino–Yano substitution model with empirical frequencies in *Splitstree4 4.16* (Huson and Bryant 2006). We also made an ordination of the samples by calculating principal components (PC) for both datasets using the PCA (principal component analysis) function in the *R* (3.6.3) package *adegenet 2.1.3* (Jombart 2008), after converting to the STRUCTURE file format using *PGDspider 2.1.1.5* (Lischer and Excoffier 2012). Finally, we analysed the genetic structure between the taxa using *sNMF* (Frichot et al. 2014) as part of the *R* package *LEA 2.6.0* (Frichot and François 2015). *sNMF* produces similar results to traditional programmes for population structure analysis, such as ADMIXTURE and STRUCTURE, but can be 10–30 times faster and has two further advantages relevant to our study system: 1) Its algorithm avoids assuming Hardy–Weinberg equilibrium (Frichot et al. 2014), likely violated in *Phyteuma* due to interspecific gene flow; and 2) the PCA‐like least squares optimisation used in *sNMF* is expected to be considerably less sensitive to uneven sampling (as present in our dataset) and hierarchical population structure (Puechmaille 2016). *sNMF* was run for 100 replications each for *K* = 1 to *K* = 10 using default settings other than enabling cross entropy, after conversion to the GENO file format using the *R* package *LEA 2.6.0* (Frichot and François 2015). The best K was determined as the one after which the cross entropy plateaus, as recommended in the manual (http://membres‐timc.imag.fr/Olivier.Francois/snmf/files/note.pdf).

As a summary statistic for coalescent modelling, we used site frequency spectra (SFS) for each deme (gene pool) as identified with *sNMF* and PCA. To avoid potential biases of the SFS from missing data, we first downsampled the 6–8 haploid genotypes (corresponding to 3–4 diploid individuals) of each sampling site by randomly sampling four (present) haploid genotypes from across all individuals for each locus within the respective sampling site, thus ensuring that no missing data are present in the dataset (similar approach as in Bagley et al. 2017, and Ye et al. 2020). Both the subsampling and the calculation of the folded 2D SFS were done using SFS scripts (https://github.com/marqueda/SFS‐scripts). The coalescent modelling was further done using *FastSimCoal 2.7* (Excoffier et al. 2021), where each model was run in 100 replicates for 100,000 coalescent simulations and 60 optimisation cycles each. Preliminary analyses of the most parameter rich models showed that the likelihood started to stabilise already after 40 cycles, thus 60 cycles are sufficient to reach stable results. All simulations were set to ignore singletons and monomorphic sites during the likelihood computation, since we filtered out singletons and by focusing on polymorphic RAD loci, we could not ensure an accurate genomic estimate of the ratio of polymorphic‐to‐monomorphic sites.

**FIGURE 2:**
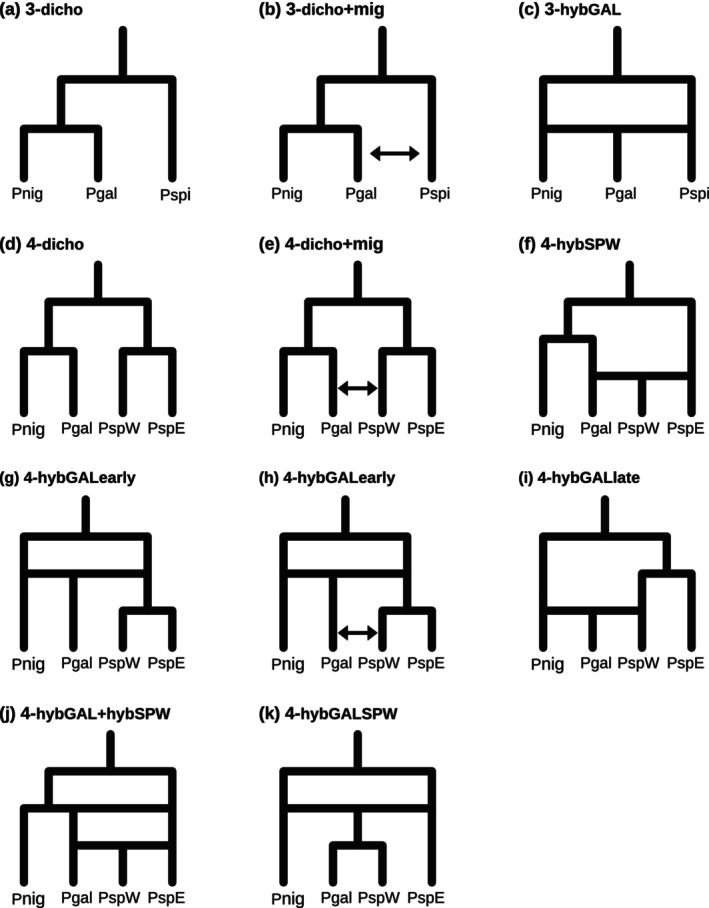
Models of the origin and evolution of *P. gallicum* tested using coalescent modelling in *FastSimCoal2*. Gene flow unique to certain models (the background gene flow as described in the running text is not shown) is indicated by bi‐directional arrows. Pgal: *Ph. gallicum*; Pnig: *Ph. nigrum*; PspE: Eastern *Ph. spicatum*; Pspi: *Ph. spicatum*; PspW: Western *Ph. spicatum*.

To take into account the high admixture of western *Ph. spicatum* (sampling sites west of Rivers Rhine and Rhone; see Results), we designed two sets of models. In the 3‐demes models, western *Ph. spicatum* is merged with eastern *Ph. spicatum* (sampling sites east of Rivers Rhine and Rhone), that is, *Ph. spicatum* is treated as a single lineage as suggested by morphology and current taxonomy (Figure 2a–c). In the 4‐demes models, western *Ph. spicatum* is treated as a separate deme (Figure 2d–k), allowing simulation of gene flow involving the two *Ph. spicatum* lineages independently. Two aspects of background gene flow were included in all models (where applicable): (i) Given that hybridisation between *Ph. spicatum* and *Ph. nigrum* is well documented (Schulz 1904; Weeda 1989; Buttler and Hand 2008; Müller et al. 2021), we allowed estimation of gene flow between the two species in all models (in the case of the 4‐demes model only between *Ph. nigrum* and eastern *Ph. spicatum*, since the range of *Ph. nigrum* does not overlap with that of western *Ph. spicatum*); and (ii) because individuals from sampling sites at the boundary between eastern and western *Ph. spicatum* were often genetically admixed (see Results), we allowed gene flow between the two groups of *Ph. spicatum* in all the 4‐demes models.

To test the dichotomous speciation hypothesis, both pure dichotomous divergence between *Ph. gallicum* and *Ph. nigrum* with no gene flow allowed (other than the previously described background gene flow; Figure 2a,d), and dichotomous divergence with subsequent gene flow between *Ph. gallicum* and *Ph. spicatum* (Figure 2b,e; only western *Ph. spicatum* in the 4‐demes models) were modelled. Due to the limitation that branching order has to be predefined in *FastSimCoal 2.7*, the 4‐demes dichotomous models (with or without gene flow: Figure 2d–e) had two divergence‐order submodels: One where *Ph. nigrum* and *Ph. gallicum* split first and eastern *Ph. spicatum* and western *Ph. spicatum* split second and another where it was the reverse. An additional 4‐demes model was included, where western *Ph. spicatum* forms as a hybrid between *Ph. gallicum* and eastern *Ph. spicatum*, but *Ph. gallicum* still splits dichotomously from *Ph. nigrum* (Figure 2f); this model was intended to test if the observed admixture in western *Ph. spicatum* could be better described by a hybrid origin, rather than just gene flow with *Ph. gallicum*.

**FIGURE 3:**
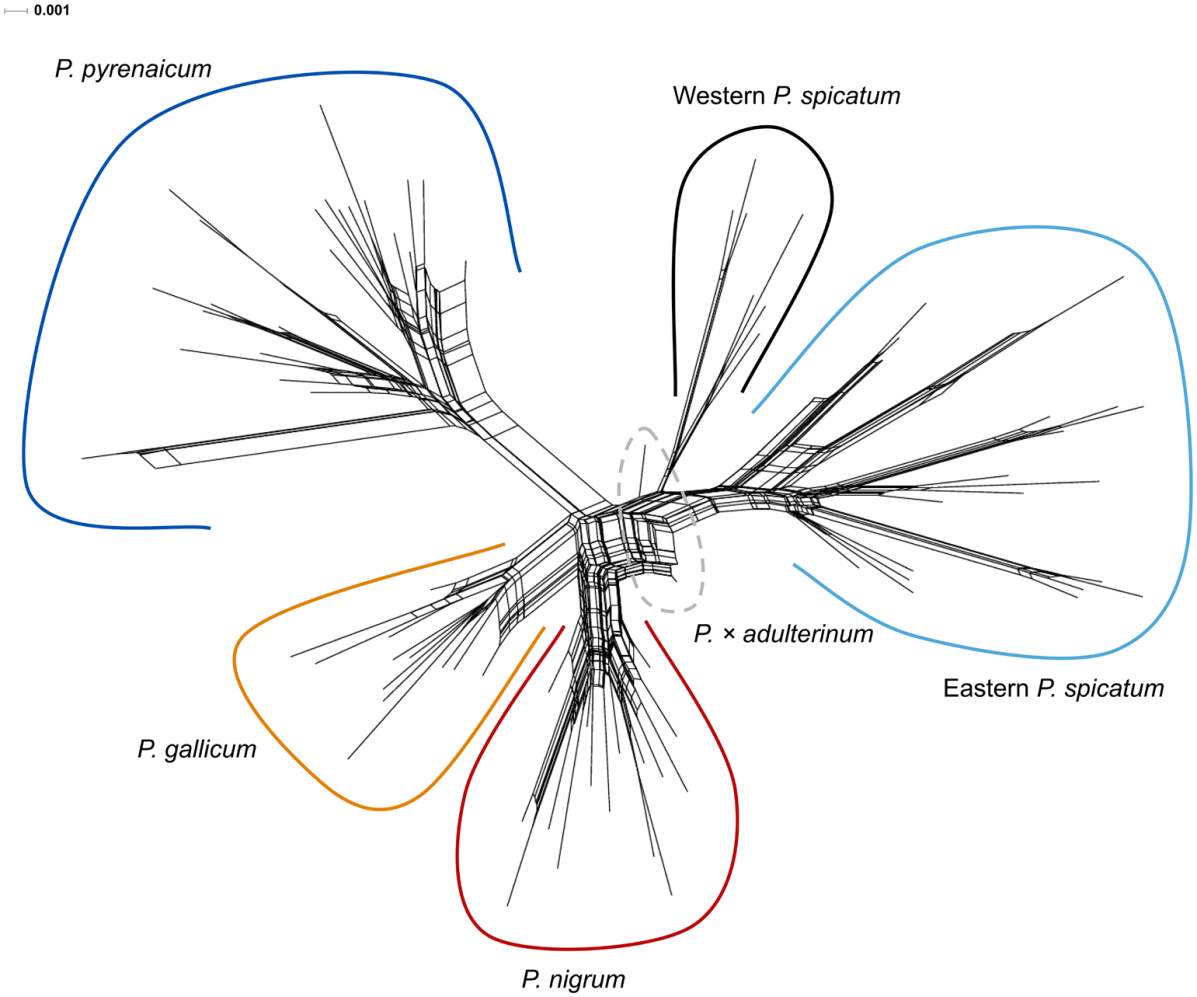
Neighbour net of the complete dataset. Branch lengths in substitutions/site.

**FIGURE 4:**
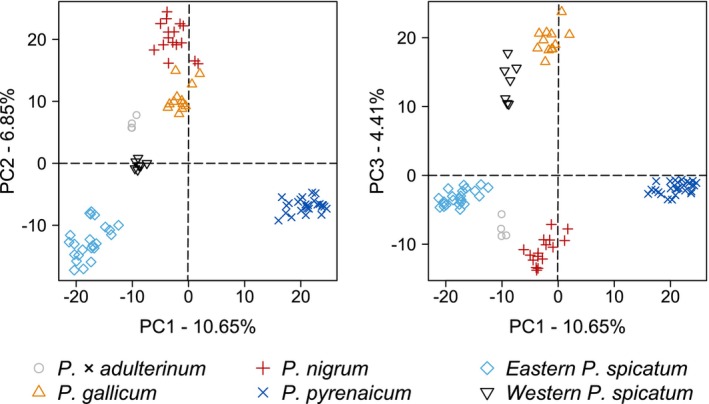
PCA plots for principal components (PC) 1 and 2 (left) and 1 and 3 (right).

The hypothesis of a hybrid origin of *Ph. gallicum* was tested using one model for the 3‐demes models (Figure 2c) and five models for the 4‐demes models to take into account different nuances in the interactions between *Ph. gallicum* and western *Ph. spicatum* (Figure 2g–j). These 4‐demes hybrid‐origin models were as follows: (1) Hybrid origin of *Ph. gallicum* with *Ph. nigrum* and the ancestor of all *Ph. spicatum* lineages as parents (Figure 2g); (2) as before, but additionally with gene flow between western *Ph. spicatum* and *Ph. gallicum* after *Ph. spicatum* splits into its two lineages (Figure 2h); (3) hybrid origin of *Ph. gallicum* with *Ph. nigrum* and western *Ph. spicatum* as parents (Figure 2i); (4) hybrid origin of *Ph. gallicum* with *Ph. nigrum* and the ancestor of all *Ph. spicatum* lineages as parents and subsequent hybrid origin of western *Ph. spicatum* with *Ph. gallicum* and eastern *Ph. spicatum* as parents (Figure 2j); and (5) *Ph. gallicum* and western *Ph. spicatum* have the same ancestor, which is of hybrid origin between *Ph. nigrum* and eastern *Ph. spicatum* (Figure 2k).

Hybridisation is expected to be associated with strong bottlenecks (Moran et al. 2021), therefore a posthybridisation bottleneck was modelled by setting the population size of the hybrid species to 0.5% of its present size for 50 generations after its formation. The choice of 50 generations for the time it takes for a new hybrid species to stabilise its genome is considered reasonable given results from studies of hybrid species formed in recent times, including *Hippophae goniocarpa* (Wang, Schluetz, and Liu 2008) and *Senecio squalidus* (Abbott et al. 2000), as well as from greenhouse experiments (Rieseberg et al. 1996; Rieseberg 2000) and modelling approaches (McCarthy, Asmussen, and Anderson 1995; Ungerer et al. 1998; Buerkle et al. 2000; but also see Buerkle and Rieseberg 2008).

In order to derive an informed interval to be used as a sampling range for the estimation of the parameter determining the divergence time of the common ancestor (TDIV_ANC_), we calculated the maximum and minimum genetic distance between *Ph. nigrum* and *Ph. spicatum*. Since including introgressed samples (i.e., western *Ph. spicatum*) may lead to a drastic underestimation of the minimum distance to the common ancestor, such samples were excluded. We extracted the full sequences of *Ph. nigrum* and eastern *Ph. spicatum* for the loci retained in the reduced dataset. Because the locus IDs are altered by *populations* when using a vcf file as input, as was done when producing the reduced dataset, a custom python script (called *extract_locusID_vcf.py* available from https://github.com/DennisLarsson/extract_locusID_vcf) was used to extract the original locus IDs from the complete dataset, by matching the SNP information between the reduced and the complete datasets. Duplicate entries have been checked for and removed manually. Based on these locus IDs, a whitelist was created, which was used with *populations* to produce full sequence information for the loci in the reduced dataset. Subsequently, *jModelTest 2.1.10* (Darriba et al. 2012) was used with default settings (number of substitution schemes was 11, unequal base frequencies were allowed and number of rate categories was 4), with the exception that we did not enable invariable sites. The GTR + G model was selected as the best model (ΔAIC of 11.69 to the second best model) and used to calculate a distance matrix in *Splitstree 4.16*. The minimum and maximum genetic distances between any pair of *Ph. nigrum* and eastern *Ph. spicatum* were extracted (0.00049 and 0.00163 substitutions/site), converted to generations using the mutation rate of *Arabidopsis thaliana* (5.9E‐09 substitutions/site/generation; Ossowski et al. 2010) and divided by 2 to get the number of generations to the common ancestor (41,922 and 138,931 generations). The values 42,000 and 139,000 were then used to set the lower and upper boundaries, respectively, of the sampling range of TDIV_ANC_. Thus, obtained divergence ages have to be interpreted with caution, as including only polymorphic RAD tags may lead to an overestimation of divergence times while sampling preferentially from coding and low‐copy sites (due to using a restriction enzyme with a GC‐rich recognition site) may lead to an underestimation of these ages.

In order to get a 95% confidence interval (CI) of the estimated age parameters (time of divergence of the ancestor of all taxa and time of origin of *Ph. gallicum*), for the best‐fitting model, we used the parametric bootstrapping approach of Ye et al. (2020). Briefly, we used *FastSimCoal 2.7* to simulate 100 pseudoreplicate SFS under the parameters of the best‐fitting run. Each of these pseudoreplicate SFS was then used as observed data for another 100 replicate analyses using the same settings as for the original data (100,000 coalescent simulations and 60 optimisation cycles). The best‐fitting replicate was then selected for each simulated SFS and used to calculate the mean parameter estimates and the 95% CIs.

We calculated the AIC score from the estimated maximum likelihood of the best run for each model in order to select the best fitting 3‐ and 4‐demes model, separately. It is important to note that because the observed data, the SFS, are different in the 3‐ and 4‐demes models, model comparisons between 3‐ and 4‐demes models are not possible. We considered the best model to be significantly better than the second‐best model if the difference in AIC scores (ΔAIC) was at least 10 (Kass and Raftery 1995).

## 3 Results

The number of retained reads per sample after demultiplexing was on average 1,085,612 (range from 569,272 to 1,786,415). Using the optimal assembly parameters (*n* = 5, *M* = 4) and a selection of accessions with the highest number of reads, de novo assembly of the artificial reference resulted in 224,417 contigs. After filtering for missingness and number of SNPs per contig, 59,888 contigs were retained. An additional 248 contigs were removed since they matched a nonspermatophyte genome, leaving 59,640 contigs in the artificial reference. After postprocessing and mapping between 401,107 and 1,208,714 reads (average 734,425 reads) of all samples to the artificial reference, 60,933 loci were assembled and used for SNP calling, resulting in 41,912 filtered SNPs for the complete dataset and 12,486 SNPs for the reduced dataset.

In the neighbour net, four groups corresponding to *Ph. pyrenaicum, Ph. nigrum, Ph. gallicum* and *Ph. spicatum* could be distinguished (Figure 3). Individuals of *Ph. × adulterinum* were positioned between *Ph. spicatum* and *Ph. nigrum*, sharing splits with both of them and having short terminal branches, as expected for a hybrid. Individuals from sampling sites of western *Ph. spicatum* were clearly separated from those of eastern *Ph. spicatum*, forming a group placed in between eastern *Ph. spicatum* and the other species, rather close to *Ph. × adulterinum*.

PCA distinguished in the first three principal components the same four major groups as the neighbour net (Figure 4). Along the first axis (explaining 10.65% of the variance), *Ph. pyrenaicum* was separated from the other species, whereas along the second axis (explaining 6.85% of the variance), *Ph. nigrum* and *Ph. gallicum* were separated from the other species; on the third axis (explaining 4.41% of the variance), *Ph. nigrum* and *Ph. gallicum* were separated from each other. *Phyteuma × adulterinum* was positioned in between eastern *Ph. spicatum* and *Ph. nigrum*, whereas western *Ph. spicatum* was positioned in between *Ph. gallicum* and eastern *Ph. spicatum*; western and eastern *Ph. spicatum* were separated from each other along the second and the third axes.

Using *K* = 4, where the cross entropy reached a plateau, *sNMF* identified four groups (Figure 1) corresponding to *Ph. pyrenaicum, Ph. nigrum, Ph. gallicum* and eastern *Ph. spicatum*. *Phyteuma × adulterinum* had a mixed ancestry between *Ph. spicatum* and *Ph. nigrum*, and western *Ph. spicatum* had a mixed ancestry between eastern *Ph. spicatum* and *Ph. gallicum*.

All models had a high fit, as in all cases the maximum estimated likelihood (Est) was close to the maximum observed likelihood (Obs; the likelihood if there were a perfect fit between the expected and the observed SFS), resulting in their ratio to be at least 0.97 (Table 2). The best fitting 3‐deme model was dichotomous divergence with subsequent gene flow between *Ph. gallicum* and *Ph. spicatum* (3‐dicho+mig), with a ΔAIC of 63.52 to the second‐best model, a hybrid origin of *Ph. gallicum* (3‐hybGAL; Table 2). The 4‐demes model that performed best was dichotomous divergence with subsequent gene flow between *Ph. gallicum* and western *Ph. spicatum* (4‐dicho+mig); the two submodels differing in the order of the splits *nigrum*–*gallicum* and western *spicatum*–eastern *spicatum*, respectively, did not differ significantly with a ΔAIC of only 0.78 (Table 2). The second best model (4‐hybGALearly+mig) with a ΔAIC difference of 21.91 to the best model was a hybrid origin of *Ph. gallicum* with *Ph. nigrum* and the ancestor of all *Ph. spicatum* lineages as parents with gene flow between western *Ph. spicatum* and *Ph. gallicum* after the split within *Ph. spicatum* (Table 2).

**TABLE 2:** ΔAIC scores for the 3‐demes and the 4‐demes models (model acronyms as in Figure 2) and the ratio between the maximum observed (Obs) and the maximum estimated (Est) likelihood; the best models are indicated in bold.

Model^a^	ΔAIC	Obs/Est likelihood
3‐dicho	651.93	0.980
**3‐dicho + mig**	**0**	**0.992**
3‐hybGAL	63.52	0.991
4‐dicho^+^	1158.98/1037.83	0.979/0.981
**4‐dicho + mig** ^+^	**0.78/0**	**0.993/0.993**
4‐hybSPW	581.19	0.986
4‐hybGALearly	346.88	0.989
4‐hybGALearly+mig	21.91	0.992
4‐hybGALlate	241.41	0.990
4‐hybGAL+hybSPW	36.86	0.992
4‐hybGALSPW	66.94	0.992

^a^ A plus (+) marks the model with two divergence‐order submodels shown in the order divergence of *P. nigrum* and *P. gallicum* first/divergence within *P. spicatum* ancestor first (see text for details).

Given that Gawalowski and Karrer (2001) observed that it takes at least 3 years for *Ph. ovatum* (a taxon nested within *Ph. spicatum*: Schneeweiss et al. 2013) to flower, we used a generation time of 3 years when converting generations into years for the divergence times (see Table 3 for divergence time in number of generations). The estimated average divergence time of *Ph. gallicum* and *Ph. nigrum* was 53,595 years ago (95% CI: 51,960–55,230 years) for the 3‐demes model and 44,544 years ago (95% CI: 43,395–45,693 years) for the 4‐demes mode (Table 3). The age of the most recent common ancestor of *Ph. spicatum*, *Ph. gallicum* and *Ph. nigrum* was 155,371 years (95% CI: 150,955–159,788 years) in the 3‐demes model and 150,916 years (95% CI: 147,406–154,427 years) in the 4‐demes model (Table 3).

**TABLE 3:** Divergence times (mean and confidence intervals) of *Ph. nigrum* and *Ph. gallicum* and of the most recent common ancestor of *Ph. spicatum, Ph. nigrum* and *Ph. gallicum*.

Clade	Divergence time as mean (confidence interval)	
	Years	Generations
*Ph. nigrum* and *Ph. gallicum*		
3‐demes model	53,595 (51,960–55,229)	17,865 (17,320–18,410)
4‐demes model	44,544 (43,395–45,692)	14,848 (14,465–15,231)
*Ph. nigrum*, *Ph. gallicum* and *Ph. spicatum*		
3‐demes model	155,371 (150,955–159,788)	51,790 (50,318–53,263)
4‐demes model	150,916 (147,406–154,427)	50,305 (49,135–51,476)

## 4 Discussion

### 4.1 A Recent Origin is a Sufficient, Although Likely Not the Sole, Cause for Range Restriction

Speciation during the (late) Quaternary is a recurrent pattern for many extant species (Kadereit and Abbott 2021). This is also the case for range‐restricted species of the Atlantic Massif Central (Koch, Wernisch, and Schmickl 2008; Konowalik et al. 2015; Jacquemin et al. 2016; Fabritzek, Griebeler, and Kadereit 2021), including *Ph. gallicum* (this study). An exclusively Quaternary origin of these species has already been suggested by Braun‐Blanquet (1931). He hypothesised this to be due to the dramatic shifts in climate experienced during the Quaternary (changes in temperature could have been as high as 10°C over a glacial cycle: Guiot et al. 1993; Strandberg et al. 2011), likely causing major range shifts, extinctions or extirpations but also opening opportunities for species from neighbouring regions to migrate into the Massif Central.

The young ages of the species appear sufficient to explain their restricted ranges. It has been suggested under the age‐and‐area hypothesis (Willis 1922; Sheth, Morueta‐Holme, and Angert 2020) that time since speciation is an important factor in determining the geographic range of a species, especially in young species since they are likely still in the process of expanding into their potential habitats. The dispersal distance of *Ph. gallicum* may be only around 10 m (maximum dispersal distance of the closely related *Ph. spicatum* in wind tunnel experiments: Maier, Emig, and Leins 1999), translating into a stepwise range expansion since its origin 15–18,000 generations (45–64,000 years) ago of about 150–180 km. While this corresponds well with the size of the actual distribution range of *Ph. gallicum*, this simplistic scenario warrants caution for several reasons. Firstly, it assumes a local origin of *Ph. gallicum*, whereas dichotomous divergence (the most likely mode of origin of *Ph. gallicum*) does not preclude a larger starting range, for example, after vicariance. Secondly, it assumes homogeneous dispersal, thus ignoring exceptional longer distance dispersal events and any range expansions/contractions induced by later cycles of glaciation. Thirdly, the difference in range size between the sister species *Ph. nigrum* and *Ph. gallicum* indicates that other factors than mere dispersal distance play an important role. One such factor may be hybridisation (with *Ph. spicatum*) preventing (further) range expansion (Chunco 2014). Hybridisation with *Ph. spicatum* is, however, also common in *Ph. nigrum* (Schulz 1904; Weeda 1989; Buttler and Hand 2008) and, therefore, cannot account for the different range sizes of *Ph. gallicum* and *Ph. nigrum*. Another factor may be a difference in dispersion rates among species, which could explain why *Ph. nigrum* and the introgressed western *Ph. spicatum* have crossed larger distances, despite *Ph. nigrum* being of the same age as and western *Ph. spicatum* being even younger than *Ph. gallicum*. There are, however, no obvious morphological differences among these three species (Schulz 1904) that could explain different dispersion rates, and accordingly, *Ph. nigrum* and *Ph. spicatum* are assigned to distance dispersal classes sharing dispersal mode (local nonspecific) and vector (unassisted), differing only in plant height (below and above 30 cm, respectively: Lososová et al. 2023; *Ph. gallicum* is unassigned). In summary, although recency of speciation is sufficient to explain the range restriction of *Ph. gallicum*, differences in range size between *Ph. gallicum* and its closest relatives suggest that the positive relationship between evolutionary age and range size is modulated by other factors, as appears to be a common trend in both plants and animals (Alzate et al. 2023).

### 4.2 Budding Speciation and Sympatry With a Closely Related Species

The best‐supported model for the origin of *Ph. gallicum* involves dichotomous divergence, as also inferred for two other endemics from the Atlantic Massif Central (*A. cebennensis*: Koch, Wernisch, and Schmickl 2008; Jacquemin et al. 2016; Novikova et al. 2016; *L. monspeliense*: Konowalik et al. 2015), whereas models invoking a hybridogenic origin (as suggested for the Massif Central lineage of *Sempervivum tectorum* : Fabritzek, Griebeler, and Kadereit 2021) are clearly rejected. During the last glaciation, the vegetation of Central Europe was tundra or cold steppe and largely forest free, which may have forced montane and temperate species, such as the common ancestor of *Ph. gallicum* and *Ph. nigrum*, mostly (or even exclusively) to mid‐elevation refugia south of 45°N (Tzedakis, Emerson, and Hewitt 2013; the low number of high‐quality occurrence data on *Ph. gallicum* prevents us from assessing this through ecological niche modelling approaches). The estimated divergence time of *Ph. gallicum* and *Ph. nigrum* (45–54,000 years ago) falls within the Middle Pleniglacial (MPG), which lasted from 24 to 59,000 years ago and was a relatively mild period compared to the colder Early Pleniglacial (EPG; 59–79,000 years ago) and Late Pleniglacial (LPG, 12–24,000 years ago; Andel and Tzedakis 1996; Tzedakis, Emerson, and Hewitt 2013). This suggests that *Ph. gallicum* and *Ph. nigrum* split when the common ancestor expanded out of an EPG refugium towards higher elevations (*Ph. gallicum*) and towards higher latitudes (*Ph. nigrum*) during the milder MPG, thus following a vicariance scenario of speciation.

In contrast to other endemics of the Atlantic Massif Central, *Ph. gallicum* is not only of more recent origin (last glaciation vs. mid‐Pleistocene) but is also unique in being the only species to currently grow in sympatry with a closely related species, *Ph. spicatum*, into which it has introgressed. Hybridisation of species is well known in *Phyteuma* (Kovanda 1981) and common in species that regularly come into contact, such as *Ph. nigrum* and *Ph. spicatum* (forming *Ph. × adulterinum*). The fact that even the northernmost population of the western lineage of *Ph. spicatum*, which is well outside the current range of *Ph. gallicum*, is introgressed suggests that the introgression occurred before *Ph. spicatum* had expanded into its full range in Western Europe. Introgression from a local into an invasive (recolonising) species, as would be the case for *Ph. gallicum* and *Ph. spicatum*, is indeed expected (Currat et al. 2008). It is unknown whether the introgression into the western lineage of *Ph. spicatum* provided it with any adaptive traits enabling occupation of novel niches (Mallet 2007; Rieseberg and Willis 2007), such as occurrence under an Atlantic climate regime.

### 4.3 Conservation Aspects

Species with restricted ranges are predicted to have an increased risk of extinction compared to species with wider niche breadth and larger range sizes (Thuiller, Lavorel, and Araújo 2005). For the Massif Central, species losses are expected to exceed 80% by 2080 under the most pessimistic scenario of climate change (Thuiller et al. 2005). Similar levels of loss have been predicted for range‐restricted Mediterranean (Casazza et al. 2014) and South African plants (Broennimann et al. 2006). The thus dire prospect for *Ph. gallicum* may be further exacerbated by land‐use change concerning meadows that are abandoned or transformed into more intensively used types of meadows, resulting in the loss of occurrences especially at lower elevations (Tasser and Tappeiner 2002; Zechmeister et al. 2003; Niedrist et al. 2009; Janeček et al. 2013). An additional threat for *Ph. gallicum* may be hybridisation with *Ph. spicatum* due to, for instance, genetic swamping or disruptions of local adaptations (Quilodrán, Montoya‐Burgos, and Currat 2020). In light of the increasing threat for *Ph. gallicum* resulting from global climate change and land‐use change and the lack of knowledge on the evolutionary consequences of gene flow with *Ph. spicatum*, *Ph. gallicum* is of considerable conservation concern in need of further studies.

## 5 Conclusion

We show that coalescent modelling, when combined with large datasets of tens of thousands of SNPs from across the genome, can be successfully used to assess the plausibility of historical factors (such as recency of speciation) for range restriction, while simultaneously discriminating different modes of speciation (dichotomous vs. hybridogenic, with or without gene flow). The recent origins of *Ph. gallicum* and other range‐restricted species in the Atlantic Massif Central support the hypothesis of Kadereit and Abbott (2021) that Quaternary climate fluctuations were instrumental in creating present‐day diversity, at least in areas heavily affected by climate changes. A feature of *Ph. gallicum*, unique at least among range‐restricted species in the Massif Central, is that it grows in sympatry with a closely related widespread species, *Ph. spicatum*, into which it introgressed. Neither the nature of this contact zone (primary vs. secondary) nor the impact on fitness of the involved species is known. Still, fitness loss in *Ph. gallicum* due to hybridisation with *Ph. spicatum* may, together with the already limited range and habitat loss due to anthropogenic climate change and land‐use change, pose serious threats to this species.

## Author Contributions

D.L. and G.M.S. contributed to the conceptualisation of the study. P.S. and D.L. acquired and processed the data. D.L. analysed the data. All authors contributed to the writing of the manuscript at all stages.

## Conflicts of Interest

The authors declare no conflicts of interest.

## Data Availability

The RAD‐sequencing data are available in the Short Read Archive under project number PRJNA1100902. Sample metadata are available in JACQ (https://www.jacq.org/) as linked in Table 1. RADseq datasets used for analyses are available in Dryad under https://doi.org/10.5061/dryad.8gtht76x2.

## References

[mec17624-bib-0001] Abbott, R. J. , J. K. James , J. A. Irwin , and H. P. Comes . 2000. “Hybrid Origin of the Oxford Ragwort *Senecio squalidus* L.” Watsonia 23: 123–138.

[mec17624-bib-0002] Alzate, A. , R. Rozzi , J. A. Velasco , et al. 2023. “The Evolutionary Age‐Range Size Relationship is Modulated by Insularity and Dispersal in Plants and Animals.” BioRxiv. 10.1101/2023.11.11.566377.

[mec17624-bib-0003] Andel, T. H. V. , and P. C. Tzedakis . 1996. “Palaeolithic Landscapes of Europe and Environs, 150,000–25,000 Years Ago: An Overview.” Quaternary Science Reviews 15: 481–500.

[mec17624-bib-0004] Andrews, K. R. , J. M. Good , M. R. Miller , G. Luikart , and P. A. Hohenlohe . 2016. “Harnessing the Power of RADseq for Ecological and Evolutionary Genomics.” Nature Reviews Genetics 17: 81–92. 10.1038/nrg.2015.28 PMC4823021 26729255

[mec17624-bib-0005] Arnold, B. , R. B. Corbett‐Detig , D. Hartl , and K. Bomblies . 2013. “RADseq Underestimates Diversity and Introduces Genealogical Biases due to Nonrandom Haplotype Sampling.” Molecular Ecology 22: 3179–3190. 23551379 10.1111/mec.12276

[mec17624-bib-0006] Bagley, R. K. , V. C. Sousa , M. L. Niemiller , and C. R. Linnen . 2017. “History, Geography and Host Use Shape Genomewide Patterns of Genetic Variation in the Redheaded Pine Sawfly ( *Neodiprion lecontei* ).” Molecular Ecology 26: 1022–1044. 28028850 10.1111/mec.13972

[mec17624-bib-0007] Bennett, K. D. 2004. “Continuing the Debate on the Role of Quaternary Environmental Change for Macroevolution.” Philosophical Transactions of the Royal Society of London. Series B: Biological Sciences 359: 295–303. 15101585 10.1098/rstb.2003.1395 PMC1693323

[mec17624-bib-0008] Bennett, K. D. 2013. “Is the Number of Species on Earth Increasing or Decreasing? Time, Chaos and the Origin of Species.” Palaeontology 56: 1305–1325.

[mec17624-bib-0009] Brandrud, M. K. , J. Baar , M. T. Lorenzo , et al. 2020. “Phylogenomic Relationships of Diploids and the Origins of Allotetraploids in *Dactylorhiza* (Orchidaceae).” Systematic Biology 69: 91–109. 31127939 10.1093/sysbio/syz035 PMC6902629

[mec17624-bib-0010] Braun‐Blanquet, J. 1931. “L'origine et le Développement des Flores Dans le Massif Central de France Avec Aperçu sur les Migrations des Flores dans l'Europe Sud‐Occidentale (Suite et fin).” Publications de la Société Linnéenne de Lyon 76: 1–109.

[mec17624-bib-0011] Broennimann, O. , W. Thuiller , G. Hughes , G. F. Midgley , J. M. R. Alkemade , and A. Guisan . 2006. “Do Geographic Distribution, Niche Property and Life Form Explain Plants' Vulnerability to Global Change?” Global Change Biology 12: 1079–1093.

[mec17624-bib-0012] Brunerye, L. 1989. “Note sur les *Phyteuma* du Groupe *spicatum* s.l. Gr de la Flore de France.” Bulletin de la Societe Botanique du Centre‐Ouest 20: 13–21.

[mec17624-bib-0013] Buerkle, C. A. , R. J. Morris , M. A. Asmussen , and L. H. Rieseberg . 2000. “The Likelihood of Homoploid Hybrid Speciation.” Heredity 84: 441–451. 10849068 10.1046/j.1365-2540.2000.00680.x

[mec17624-bib-0014] Buerkle, C. A. , and L. H. Rieseberg . 2008. “The Rate of Genome Stabilization in Homoploid Hybrid Species.” Evolution 62: 266–275. 18039323 10.1111/j.1558-5646.2007.00267.x PMC2442919

[mec17624-bib-0015] Buttler, K. P. , and R. Hand . 2008. “Beiträge zur Fortschreibung der Florenliste Deutschlands (Pteridophyta, Spermatophyta) Zweite Folge.” Kochia 3: 75–86.

[mec17624-bib-0016] Camacho, C. , G. Coulouris , V. Avagyan , et al. 2009. “BLAST+: Architecture and Applications.” BMC Bioinformatics 10: 421. 20003500 10.1186/1471-2105-10-421 PMC2803857

[mec17624-bib-0017] Cardillo, M. , R. Dinnage , and W. McAlister . 2019. “The Relationship Between Environmental Niche Breadth and Geographic Range Size Across Plant Species.” Journal of Biogeography 46: 97–109.

[mec17624-bib-0018] Casazza, G. , P. Giordani , R. Benesperi , et al. 2014. “Climate Change Hastens the Urgency of Conservation for Range‐Restricted Plant Species in the Central‐Northern Mediterranean Region.” Biological Conservation 179: 129–138.

[mec17624-bib-0019] Casazza, G. , F. Grassi , G. Zecca , M. G. Mariotti , M. Guerrina , and L. Minuto . 2013. “Phylogeography of *Primula Allionii* (Primulaceae), a Narrow Endemic of the Maritime Alps.” Botanical Journal of the Linnean Society 173: 637–653.

[mec17624-bib-0020] Chunco, A. J. 2014. “Hybridization in a Warmer World.” Ecology and Evolution 4: 2019–2031. 24963394 10.1002/ece3.1052 PMC4063493

[mec17624-bib-0021] Coppi, A. , A. Mengoni , and F. Selvi . 2008. “AFLP Fingerprinting of *Anchusa* (Boraginaceae) in the Corso‐Sardinian System: Genetic Diversity, Population Differentiation and Conservation Priorities in an Insular Endemic Group Threatened With Extinction.” Biological Conservation 141: 2000–2011.

[mec17624-bib-0022] Currat, M. , M. Ruedi , R. J. Petit , and L. Excoffier . 2008. “The Hidden Side of Invasions: Massive Introgression by Local Genes.” Evolution 62: 1908–1920. 18452573 10.1111/j.1558-5646.2008.00413.x

[mec17624-bib-0023] Damboldt, J. 1976. “ *Phyteuma* L.” In Flora Europaea 4, edited by T. G. Tutin , V. H. Heywood , N. A. Burges , et al., 95–98. Cambridge, UK: Cambridge University Press.

[mec17624-bib-0024] Danecek, P. , A. Auton , G. Abecasis , et al. 2011. “The Variant Call Format and VCFtools.” Bioinformatics 27: 2156–2158. 21653522 10.1093/bioinformatics/btr330 PMC3137218

[mec17624-bib-0025] Darriba, D. , G. L. Taboada , R. Doallo , and D. Posada . 2012. “jModelTest 2: More Models, New Heuristics and Parallel Computing.” Nature Methods 9: 772. 10.1038/nmeth.2109 PMC4594756 22847109

[mec17624-bib-0026] Davey, J. L. , and M. W. Blaxter . 2010. “RADseq: Next‐Generation Population Genetics.” Briefings in Functional Genomics 9: 416–423. 21266344 10.1093/bfgp/elq031 PMC3080771

[mec17624-bib-0027] Douglas, N. A. , W. A. Wall , Q. Y. Xiang , et al. 2011. “Recent Vicariance and the Origin of the Rare, Edaphically Specialized Sandhills Lily, *Lilium pyrophilum* (Liliaceae): Evidence From Phylogenetic and Coalescent Analyses.” Molecular Ecology 20: 2901–2915. 21672067 10.1111/j.1365-294X.2011.05151.x

[mec17624-bib-0028] Emerson, K. J. , C. R. Merz , J. M. Catchen , et al. 2010. “Resolving Postglacial Phylogeography Using High‐Throughput Sequencing.” Proceedings of the National Academy of Sciences of the United States of America 107: 16196–16200. 20798348 10.1073/pnas.1006538107 PMC2941283

[mec17624-bib-0029] Escudero, M. , M. Lovit , B. H. Brown , and A. L. Hipp . 2019. “Rapid Plant Speciation Associated With the Last Glacial Period: Reproductive Isolation and Genetic Drift in Sedges.” Botanical Journal of the Linnean Society 190: 303–314.

[mec17624-bib-0030] Espeland, E. K. , and T. M. Emam . 2011. “The Value of Structuring Rarity: The Seven Types and Links to Reproductive Ecology.” Biodiversity and Conservation 20: 963–985.

[mec17624-bib-0031] Excoffier, L. , N. Marchi , D. A. Marques , R. Matthey‐Doret , A. Gouy , and V. C. Sousa . 2021. “fastsimcoal2: Demographic Inference Under Complex Evolutionary Scenarios.” Bioinformatics 37: 4882–4885. 34164653 10.1093/bioinformatics/btab468 PMC8665742

[mec17624-bib-0032] Fabritzek, A. G. , E. M. Griebeler , and J. W. Kadereit . 2021. “Hybridization, Ecogeographical Displacement and the Emergence of New Lineages—A Genotyping‐by‐Sequencing and Ecological Niche and Species Distribution Modelling Study of *Sempervivum tectorum* L. (Houseleek).” Journal of Evolutionary Biology 34: 830–844. 33714223 10.1111/jeb.13784

[mec17624-bib-0033] Fernández‐Mazuecos, M. , and P. Vargas . 2013. “Congruence Between Distribution Modelling and Phylogeographical Analyses Reveals Quaternary Survival of a Toadflax Species (*Linaria Elegans*) in Oceanic Climate Areas of a Mountain Ring Range.” New Phytologist 198: 1274–1289. 23496320 10.1111/nph.12220

[mec17624-bib-0034] Frichot, E. , and O. François . 2015. “LEA: An R Package for Landscape and Ecological Association Studies.” Methods in Ecology and Evolution 6: 925–929.

[mec17624-bib-0035] Frichot, E. , F. Mathieu , T. Trouillon , G. Bouchard , and O. François . 2014. “Fast and Efficient Estimation of Individual Ancestry Coefficients.” Genetics 196: 973–983. 24496008 10.1534/genetics.113.160572 PMC3982712

[mec17624-bib-0036] Gargiulo, R. , O. D. Castro , E. D. Guacchio , and P. Caputo . 2019. “Genetic Diversity and Origin of the Rare, Narrow Endemic *Asperula crassifolia* (Rubiaceae).” Plant Systematics and Evolution 305: 181–192.

[mec17624-bib-0037] Gaston, K. J. , and W. E. Kunin . 1997. “Rare—Common Differences: An Overview.” In The Biology of Rarity: Causes and Consequences of Rare—Common Differences, edited by W. E. Kunin and K. J. Gaston , 12–29. Dordrecht, the Netherlands: Springer Netherlands.

[mec17624-bib-0038] Gawalowski, G. , and G. Karrer . 2001. “Über die Wuchsform von *Phyteuma ovatum* (Campanulaceae).” Neilreichia 1: 165–175.

[mec17624-bib-0039] Gibson, L. , A. McNeill , P. de Tores , A. Wayne , and C. Yates . 2010. “Will Future Climate Change Threaten a Range Restricted Endemic Species, the Quokka ( *Setonix brachyurus* ), in South West Australia?” Biological Conservation 143: 2453–2461.

[mec17624-bib-0040] González‐Martínez, S. C. , K. Ridout , and J. R. Pannell . 2017. “Range Expansion Compromises Adaptive Evolution in an Outcrossing Plant.” Current Biology 27: 2544–2551. 28803874 10.1016/j.cub.2017.07.007

[mec17624-bib-0041] Grünig, S. , M. Fischer , and C. Parisod . 2021. “Recent Hybrid Speciation at the Origin of the Narrow Endemic *Pulmonaria helvetica* .” Annals of Botany 127: 21–31. 32738145 10.1093/aob/mcaa145 PMC7750729

[mec17624-bib-0042] Guiot, J. , J. L. de Beaulieu , R. Cheddadi , F. David , P. Ponel , and M. Reille . 1993. “The Climate in Western Europe During the Last Glacial/Interglacial Cycle Derived From Pollen and Insect Remains.” Palaeogeography, Palaeoclimatology, Palaeoecology 103: 73–93.

[mec17624-bib-0043] Han, E. , J. S. Sinsheimer , and J. Novembre . 2014. “Characterizing Bias in Population Genetic Inferences From Low‐Coverage Sequencing Data.” Molecular Biology and Evolution 31: 723–735. 24288159 10.1093/molbev/mst229 PMC3935184

[mec17624-bib-0044] Hohenlohe, P. A. , S. Bassham , P. D. Etter , N. Stiffler , E. A. Johnson , and W. A. Cresko . 2010. “Population Genomics of Parallel Adaptation in Threespine Stickleback Using Sequenced RAD Tags.” PLoS Genetics 6: e1000862. 20195501 10.1371/journal.pgen.1000862 PMC2829049

[mec17624-bib-0045] Huson, D. H. , and D. Bryant . 2006. “Application of Phylogenetic Networks in Evolutionary Studies.” Molecular Biology and Evolution 23: 254–267. 16221896 10.1093/molbev/msj030

[mec17624-bib-0046] Jacquemin, J. , N. Hohmann , M. Buti , et al. 2016. “Levels and Patterns of Genetic Diversity Differ Between Two Closely Related Endemic *Arabidopsis* Species.” BioRxiv. 10.1101/048785.

[mec17624-bib-0047] Janeček, Š. , F. de Bello , J. Horník , et al. 2013. “Effects of Land‐Use Changes on Plant Functional and Taxonomic Diversity Along a Productivity Gradient in Wet Meadows.” Journal of Vegetation Science 24: 898–909.

[mec17624-bib-0048] Jombart, T. 2008. “Adegenet: A R Package for the Multivariate Analysis of Genetic Markers.” Bioinformatics 24: 1403–1405. 18397895 10.1093/bioinformatics/btn129

[mec17624-bib-0049] Kadereit, J. W. , and R. J. Abbott . 2021. “Plant Speciation in the Quaternary.” Plant Ecology and Diversity 14: 105–142.

[mec17624-bib-0050] Karron, J. D. 1987. “The Pollination Ecology of Co‐Occurring Geographically Restricted and Widespread Species of *Astragalus* (Fabaceae).” Biological Conservation 39: 179–193.

[mec17624-bib-0051] Kass, R. E. , and A. E. Raftery . 1995. “Bayes Factors.” Journal of the American Statistical Association 90: 773–795.

[mec17624-bib-0052] Knowles, L. L. , and W. Maddison . 2002. “Statistical Phylogeography.” Molecular Ecology 11: 2623–2635. 12453245 10.1046/j.1365-294x.2002.01637.x

[mec17624-bib-0053] Koch, M. A. , M. Wernisch , and R. Schmickl . 2008. “ *Arabidopsis Thaliana* 's Wild Relatives: An Updated Overview on Systematics, Taxonomy and Evolution.” Taxon 57: 933–943.

[mec17624-bib-0054] Konowalik, K. , F. Wagner , S. Tomasello , R. Vogt , and C. Oberprieler . 2015. “Detecting Reticulate Relationships Among Diploid *Leucanthemum* Mill. (Compositae, Anthemideae) Taxa Using Multilocus Species Tree Reconstruction Methods and AFLP Fingerprinting.” Molecular Phylogenetics and Evolution 92: 308–328. 26103001 10.1016/j.ympev.2015.06.003

[mec17624-bib-0055] Kovanda, M. 1981. “Studies in *Phyteuma* .” Preslia 53: 211–238.

[mec17624-bib-0056] Langmead, B. , and S. L. Salzberg . 2012. “Fast Gapped‐Read Alignment With Bowtie 2.” Nature Methods 9: 357–359. 22388286 10.1038/nmeth.1923 PMC3322381

[mec17624-bib-0057] Larsson, D. J. , D. Pan , and G. M. Schneeweiss . 2021. “Addressing Alpine Plant Phylogeography Using Integrative Distributional, Demographic and Coalescent Modeling.” Alpine Botany 132: 5–19. 35368907 10.1007/s00035-021-00263-w PMC8933363

[mec17624-bib-0059] Lavergne, S. , J. D. Thompson , E. Garnier , and M. Debussche . 2004. “The Biology and Ecology of Narrow Endemic and Widespread Plants: A Comparative Study of Trait Variation in 20 Congeneric Pairs.” Oikos 107: 505–518.

[mec17624-bib-0060] Lischer, H. E. L. , and L. Excoffier . 2012. “PGDSpider: An Automated Data Conversion Tool for Connecting Population Genetics and Genomics Programs.” Bioinformatics 28: 298–299. 22110245 10.1093/bioinformatics/btr642

[mec17624-bib-0061] Liu, W. , J. Xie , H. Zhou , et al. 2021. “Population Dynamics Linked to Glacial Cycles in *Cercis chuniana* F. P. Metcalf (Fabaceae) Endemic to the Montane Regions of Subtropical China.” Evolutionary Applications 14: 2647–2663. 34815745 10.1111/eva.13301 PMC8591333

[mec17624-bib-0062] Lososová, Z. , I. Axmanová , M. Chytrý , et al. 2023. “Seed Dispersal Distance Classes and Dispersal Modes for the European Flora.” Global Ecology and Biogeography 32: 1485–1494.

[mec17624-bib-0063] Lu, K. , L. Wei , X. Li , et al. 2019. “Whole‐Genome Resequencing Reveals *Brassica napus* Origin and Genetic Loci Involved in Its Improvement.” Nature Communications 10: 1154. 10.1038/s41467-019-09134-9 PMC6411957 30858362

[mec17624-bib-0064] Lu, R. S. , Y. Chen , I. Tamaki , et al. 2020. “Pre‐Quaternary Diversification and Glacial Demographic Expansions of *Cardiocrinum* (Liliaceae) in Temperate Forest Biomes of Sino‐Japanese Floristic Region.” Molecular Phylogenetics and Evolution 143: 106693. 31778814 10.1016/j.ympev.2019.106693

[mec17624-bib-0065] Maier, A. , W. Emig , and P. Leins . 1999. “Dispersal Patterns of Some *Phyteuma* Species (Campanulaceae).” Plant Biology 1: 408–417.

[mec17624-bib-0066] Mallet, J. 2007. “Hybrid Speciation.” Nature 446: 279–283. 17361174 10.1038/nature05706

[mec17624-bib-0067] Marcussen, T. , K. Blaxland , M. D. Windham , K. E. Haskins , and F. Armstrong . 2011. “Establishing the Phylogenetic Origin, History, and Age of the Narrow Endemic *Viola guadalupensis* (Violaceae).” American Journal of Botany 98: 1978–1988. 22081412 10.3732/ajb.1100208

[mec17624-bib-0068] Mayol, M. , C. Palau , J. A. Rosselló , S. C. González‐Marítnez , A. Molins , and M. Riba . 2012. “Patterns of Genetic Variability and Habitat Occupancy in *Crepis triasii* (Asteraceae) at Different Spatial Scales: Insights on Evolutionary Processes Leading to Diversification in Continental Islands.” Annals of Botany 109: 429–441. 22167790 10.1093/aob/mcr298 PMC3268543

[mec17624-bib-0069] McCarthy, E. M. , M. A. Asmussen , and W. W. Anderson . 1995. “A Theoretical Assessment of Recombinational Speciation.” Heredity 74: 502–509.

[mec17624-bib-0070] McKenna, A. , M. Hanna , E. Banks , et al. 2010. “The Genome Analysis Toolkit: A MapReduce Framework for Analyzing Next‐Generation DNA Sequencing Data.” Genome Research 20: 1297–1303. 20644199 10.1101/gr.107524.110 PMC2928508

[mec17624-bib-0071] Meusel, H. , and E. Jäger . 1992. Vergleichende Chorologie der Zentraleuropäischen Flora 3. Jena, Germany: Gustav Fischer.

[mec17624-bib-0072] MNHN, OFB . 2023. “National Inventory of Natural Heritage (INPN).” https://inpn.mnhn.fr/.

[mec17624-bib-0073] Moran, B. M. , C. Payne , Q. Langdon , D. L. Powell , Y. Brandvain , and M. Schumer . 2021. “The Genomic Consequences of Hybridization.” eLife 10: e69016. 34346866 10.7554/eLife.69016 PMC8337078

[mec17624-bib-0074] Müller, F. , C. M. Ritz , E. Welk , and K. Wesche . 2021. Rothmaler—Exkursionsflora von Deutschland. Gefäßpflanzen: Grundband, 22nd edition. Berlin, Germany: Springer.

[mec17624-bib-0075] Murray, B. R. , P. H. Thrall , A. M. Gill , and A. B. Nicotra . 2002. “How Plant Life‐History and Ecological Traits Relate to Species Rarity and Commonness at Varying Spatial Scales.” Austral Ecology 27: 291–310.

[mec17624-bib-0076] Nevado, B. , N. Contreras‐Ortiz , C. Hughes , and D. A. Filatov . 2018. “Pleistocene Glacial Cycles Drive Isolation, Gene Flow and Speciation in the High‐Elevation Andes.” New Phytologist 219: 779–793. 29862512 10.1111/nph.15243

[mec17624-bib-0077] Niedrist, G. , E. Tasser , C. Lüth , J. D. Via , and U. Tappeiner . 2009. “Plant Diversity Declines With Recent Land Use Changes in European Alps.” Plant Ecology 202: 195–210.

[mec17624-bib-0078] Novikova, P. Y. , N. Hohmann , V. Nizhynska , et al. 2016. “Sequencing of the Genus *Arabidopsis* Identifies a Complex History of Nonbifurcating Speciation and Abundant Trans‐Specific Polymorphism.” Nature Genetics 48: 1077–1082. 27428747 10.1038/ng.3617

[mec17624-bib-0079] O'Leary, S. J. , J. B. Puritz , S. C. Willis , C. M. Hollenbeck , and D. S. Portnoy . 2018. “These Aren't the Loci You're Looking for: Principles of Effective SNP Filtering for Molecular Ecologists.” Molecular Ecology 27: 3193–3206. 10.1111/mec.14792 29987880

[mec17624-bib-0080] Otero, A. , M. Fernández‐Mazuecos , and P. Vargas . 2021. “Evolution in the Model Genus *Antirrhinum* Based on Phylogenomics of Topotypic Material.” Frontiers in Plant Science 12: 631178. 33643359 10.3389/fpls.2021.631178 PMC7907437

[mec17624-bib-0081] Paris, J. R. , J. R. Stevens , and J. M. Catchen . 2017. “Lost in Parameter Space: A Road Map for Stacks.” Methods in Ecology and Evolution 8: 1360–1373.

[mec17624-bib-0082] Paun, O. , B. Turner , E. Trucchi , J. Munzinger , M. W. Chase , and R. Samuel . 2016. “Processes Driving the Adaptive Radiation of a Tropical Tree (*Diospyros*, Ebenaceae) in New Caledonia, a Biodiversity Hotspot.” Systematic Biology 65: 212–227. 26430059 10.1093/sysbio/syv076 PMC4748748

[mec17624-bib-0083] Pouget, M. , S. Youssef , J. Migliore , M. Juin , F. Médail , and A. Baumel . 2013. “Phylogeography Sheds Light on the Central–Marginal Hypothesis in a Mediterranean Narrow Endemic Plant.” Annals of Botany 112: 1409–1420. 23962409 10.1093/aob/mct183 PMC3806523

[mec17624-bib-0084] Puechmaille, S. J. 2016. “The Program structure Does Not Reliably Recover the Correct Population Structure When Sampling Is Uneven: Subsampling and New Estimators Alleviate the Problem.” Molecular Ecology Resources 16: 608–627. 26856252 10.1111/1755-0998.12512

[mec17624-bib-0085] Quilodrán, C. S. , J. I. Montoya‐Burgos , and M. Currat . 2020. “Harmonizing Hybridization Dissonance in Conservation.” Communications Biology 3: 391. 32694629 10.1038/s42003-020-1116-9 PMC7374702

[mec17624-bib-0086] Rieseberg, L. H. 2000. “Crossing Relationships Among Ancient and Experimental Sunflower Hybrid Lineages.” Evolution 54: 859–865. 10937259 10.1111/j.0014-3820.2000.tb00086.x

[mec17624-bib-0087] Rieseberg, L. H. , B. Sinervo , C. R. Linder , M. C. Ungerer , and D. M. Arias . 1996. “Role of Gene Interactions in Hybrid Speciation: Evidence From Ancient and Experimental Hybrids.” Science 272: 741–745. 8662570 10.1126/science.272.5262.741

[mec17624-bib-0088] Rieseberg, L. H. , and J. H. Willis . 2007. “Plant Speciation.” Science 317: 910–914. 17702935 10.1126/science.1137729 PMC2442920

[mec17624-bib-0089] Rochette, N. C. , A. G. Rivera‐Colón , and J. M. Catchen . 2019. “Stacks 2: Analytical Methods for Paired‐End Sequencing Improve RADseq‐Based Population Genomics.” Molecular Ecology 28: 4737–4754. 31550391 10.1111/mec.15253

[mec17624-bib-0090] Sales, F. , and I. C. Hedge . 2000. “ *Phyteuma* L.(Campanulaceae)—Some Taxonomic Notes.” Anales del Jardin Botánico de Madrid 57: 474–477.

[mec17624-bib-0091] Schneeweiss, G. M. , C. Pachschwöll , A. Tribsch , et al. 2013. “Molecular Phylogenetic Analyses Identify Alpine Differentiation and Dysploid Chromosome Number Changes as Major Forces for the Evolution of the European Endemic *Phyteuma* (Campanulaceae).” Molecular Phylogenetics and Evolution 69: 634–652. 23891952 10.1016/j.ympev.2013.07.015

[mec17624-bib-0092] Schulz, R. 1904. Monographie der Gattung Phyteuma. Geisenheim a. Rh., Germany: J. Schneck.

[mec17624-bib-0093] Shang, H. Y. , Z. H. Li , M. Dong , et al. 2015. “Evolutionary Origin and Demographic History of an Ancient Conifer (*Juniperus microsperma*) in the Qinghai‐Tibetan Plateau.” Scientific Reports 5: 10216. 25977142 10.1038/srep10216 PMC4432373

[mec17624-bib-0094] Sheth, S. N. , N. Morueta‐Holme , and A. L. Angert . 2020. “Determinants of Geographic Range Size in Plants.” New Phytologist 226: 650–665. 31901139 10.1111/nph.16406

[mec17624-bib-0095] Simurda, M. C. , D. C. Marshall , and J. S. Knox . 2005. “Phylogeography of the Narrow Endemic, *Helenium virginicum* (Asteraceae), based Upon ITS Sequence Comparisons.” Systematic Botany 30: 887–898.

[mec17624-bib-0096] Strandberg, G. , J. Brandefelt , E. Kjellström , and B. Smith . 2011. “High‐Resolution Regional Simulation of Last Glacial Maximum Climate in Europe.” Tellus A: Dynamic Meteorology and Oceanography 63A: 107–125.

[mec17624-bib-0097] Tasser, E. , and U. Tappeiner . 2002. “Impact of Land Use Changes on Mountain Vegetation.” Applied Vegetation Science 5: 173–184.

[mec17624-bib-0098] Tela Botanica . 2023. “*Phyteuma gallicum—ecologie—eFlore—Tela Botanica* .” https://www.tela‐botanica.org/bdtfx‐nn‐49211‐ecologie/.

[mec17624-bib-0099] Thuiller, W. , S. Lavorel , and M. B. Araújo . 2005. “Niche Properties and Geographical Extent as Predictors of Species Sensitivity to Climate Change.” Global Ecology and Biogeography 14: 347–357.

[mec17624-bib-0100] Thuiller, W. , S. Lavorel , M. B. Araújo , M. T. Sykes , I. C. Prentice , and H. A. Mooney . 2005. “Climate Change Threats to Plant Diversity in Europe.” Proceedings of the National Academy of Sciences of the United States of America 102: 8245–8250. 15919825 10.1073/pnas.0409902102 PMC1140480

[mec17624-bib-0101] Tison, J.‐M. , and B. de Foucault . 2014. Flora Gallica: Flore de France. Mèze, France: Biotope.

[mec17624-bib-0102] Tournebize, R. , S. Manel , Y. Vigouroux , F. Munoz , A. D. Kochko , and V. Poncet . 2017. “Two Disjunct Pleistocene Populations and Anisotropic Postglacial Expansion Shaped the Current Genetic Structure of the Relict Plant *Amborella Trichopoda* .” PLoS One 12: e0183412. 28820899 10.1371/journal.pone.0183412 PMC5562301

[mec17624-bib-0103] Tzedakis, P. C. , B. C. Emerson , and G. M. Hewitt . 2013. “Cryptic or Mystic? Glacial Tree Refugia in Northern Europe.” Trends in Ecology & Evolution 28: 696–704. 24091207 10.1016/j.tree.2013.09.001

[mec17624-bib-0104] Ungerer, M. C. , S. J. E. Baird , J. Pan , and L. H. Rieseberg . 1998. “Rapid Hybrid Speciation in Wild Sunflowers.” Proceedings of the National Academy of Sciences of the United States of America 95: 11757–11762. 9751738 10.1073/pnas.95.20.11757 PMC21713

[mec17624-bib-0105] Vargas, O. M. , B. Goldston , D. L. Grossenbacher , and K. M. Kay . 2020. “Patterns of Speciation Are Similar Across Mountainous and Lowland Regions for a Neotropical Plant Radiation (Costaceae: *Costus*).” Evolution 74: 2644–2661. 33047821 10.1111/evo.14108

[mec17624-bib-0106] Wang, A. , F. Schluetz , and J. Liu . 2008. “Molecular Evidence for Double Maternal Origins of the Diploid Hybrid *Hippophae goniocarpa* (Elaeagnaceae).” Botanical Journal of the Linnean Society 156: 111–118.

[mec17624-bib-0107] Weeda, E. J. 1989. “ *Phyteuma nigrum* F.W. Schmidt en *P. spicatum* L. in Nederland.” Gorteria Dutch Botanical Archives 15: 6–27.

[mec17624-bib-0108] Willis, J. C. 1922. Area and Area: A Study in Geographical Distribution and Origin of Species. Cambridge, UK: Cambridge University Press.

[mec17624-bib-0109] Willis, K. J. , and K. J. Niklas . 2004. “The Role of Quaternary Environmental Change in Plant Macroevolution: The Exception or the Rule?” Philosophical Transactions of the Royal Society, B: Biological Sciences 359: 159–172. 10.1098/rstb.2003.1387 PMC1693324 15101573

[mec17624-bib-0110] Ye, Z. , D. Chen , J. Yuan , et al. 2020. “Are Population Isolations and Declines a Threat to Island Endemic Water Striders? A Lesson From Demographic and Niche Modelling of *Metrocoris esakii* (Hemiptera: Gerridae).” Molecular Ecology 29: 4573–4587. 33006793 10.1111/mec.15669

[mec17624-bib-0111] Zechmeister, H. G. , I. Schmitzberger , B. Steurer , J. Peterseil , and T. Wrbka . 2003. “The Influence of Land‐Use Practices and Economics on Plant Species Richness in Meadows.” Biological Conservation 114: 165–177.

